# Chemistry and Biochemistry of Sulfur Natural Compounds: Key Intermediates of Metabolism and Redox Biology

**DOI:** 10.1155/2020/8294158

**Published:** 2020-09-29

**Authors:** Antonio Francioso, Alessia Baseggio Conrado, Luciana Mosca, Mario Fontana

**Affiliations:** ^1^Department of Biochemical Sciences “A. Rossi Fanelli”, Sapienza University of Rome, 00185 Rome, Italy; ^2^Department of Organic Chemistry, Instituto Universitario de Bio-Orgánica Antonio González, University of La Laguna, La Laguna, 38296 Tenerife, Spain

## Abstract

Sulfur contributes significantly to nature chemical diversity and thanks to its particular features allows fundamental biological reactions that no other element allows. Sulfur natural compounds are utilized by all living beings and depending on the function are distributed in the different kingdoms. It is no coincidence that marine organisms are one of the most important sources of sulfur natural products since most of the inorganic sulfur is metabolized in ocean environments where this element is abundant. Terrestrial organisms such as plants and microorganisms are also able to incorporate sulfur in organic molecules to produce primary metabolites (e.g., methionine, cysteine) and more complex unique chemical structures with diverse biological roles. Animals are not able to fix inorganic sulfur into biomolecules and are completely dependent on preformed organic sulfurous compounds to satisfy their sulfur needs. However, some higher species such as humans are able to build new sulfur-containing chemical entities starting especially from plants' organosulfur precursors. Sulfur metabolism in humans is very complicated and plays a central role in redox biochemistry. The chemical properties, the large number of oxidation states, and the versatile reactivity of the oxygen family chalcogens make sulfur ideal for redox biological reactions and electron transfer processes. This review will explore sulfur metabolism related to redox biochemistry and will describe the various classes of sulfur-containing compounds spread all over the natural kingdoms. We will describe the chemistry and the biochemistry of well-known metabolites and also of the unknown and poorly studied sulfur natural products which are still in search for a biological role.

## 1. Introduction

In living organisms, sulfur is one of the most fundamental elements and the seventh most abundant mineral in the human body. Sulfur belongs to chalcogens, elements of the 16 group of the periodic table, which display the awesome characteristic of having a variety of redox states and redox potentials allowing them to form interchalcogen bonds and atom exchange reactions, giving rise to a vast number of sulfur species that take part in biological processes. Noteworthy, the bulk of biomolecules consists only of carbon, hydrogen, nitrogen, and oxygen atoms, and the presence of sulfur accounts for the distinctive properties of sulfur compounds. Actually, sulfur and oxygen belong to the same group in the periodic table; however, Met and Cys analogues with the sulfur atom replaced by oxygen do not serve the same function. Sulfur has unique characteristics that differentiate it from oxygen. The increased atomic size confers to sulfur a lower electronegativity than oxygen. The thioether (R_2_S) moiety of Met is more reactive than the analogue ether (R_2_O). Thioethers can form sulfonium ions (R_3_S^+^) by donating electrons to other organic species thanks to their ability to sink electrons and stabilize a negative charge on a neighboring carbon [1]. These compounds undergo sequential oxidation to sulfoxides (R_2_SO) and sulfones (R_2_SO_2_), conferring to these derivatives novel unexpected roles. In cell metabolism, a sulfonium compound such as S-adenosylmethionine (SAM) mediates most biochemical methylation reactions. It is doubtful whether other amino acid derivatives or other “-onium” compounds could play this role: quaternary ammonium compounds are unable to effectively methylate acceptor compounds, and oxonium compounds, such as a hypothetical oxygen analogue of SAM, would produce such a powerful methylating agent that it would methylate cellular nucleophiles without the need for an enzyme [2, 3]. The sulfur compounds contained in food are amino acids or vitamins including methionine (Met), cysteine (Cys), homocysteine (HCy), cystine (Cys-Cys), taurine (Tau), lipoic acid, thiamine, and biotin as well as the glucosinolates and allylic sulfur compounds that are contained in cabbage and cauliflower (cruciferous vegetables). The amount of sulfur compounds in food greatly varies depending on the type of food: 8% for egg white, 5% for beef as well as for chicken and fish, and 4% for dairy products and plant proteins [4]. The recommended dietary allowance (RDA) for sulfur has been estimated to be 13-14 mg/kg of body weight per day. Considering 70 kg weight for a person, not affected by sex or age, this means 1.1 g of sulfur per day [5–9]. Among the sulfur compounds ingested with food, Met and Cys represent the largest part and are extensively metabolized by the organisms [3]. The Met/Cys ratio in food is 3/1 for dairy products, fish, and meat and 4/3 for eggs and plant products such as soybeans [10, 11]. Met is an essential amino acid assumed by diet and cannot be synthesized contrary to nonessential Cys. Numerous key metabolic intermediates such as HCy, Cys-Cys, and Tau are generated by these sulfur amino acids [4, 12]. Throughout the transsulfuration pathway, Met can be converted to Cys with a yield depending on cell needs. Interestingly, both these two sulfur amino acids cannot be stored as such in the body but cysteine can be stocked as glutathione (GSH) and sulfur excess is promptly excreted in the urine after its oxidation to sulfate or reabsorbed if required [13].

In this review, we will explore the fundamental aspects of sulfur metabolism and redox biochemistry and the large pool of naturally occurring sulfur-containing compounds. We will focus on the chemistry and the biochemistry of exogenous and endogenous sulfur metabolites. We will underline the importance of well-known and widely studied molecules, and we will also focus on unknown and poorly studied sulfur natural products whose biological role is still a mystery and needs to be investigated [14, 15].

## 2. How Sulfur Comes to “Life”

**Figure 1:**
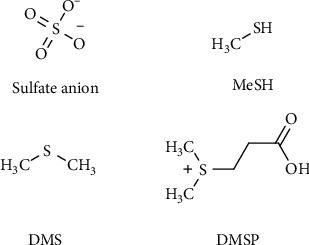
Inorganic and organic forms of entry for sulfur into biosynthetic metabolism of plants and microorganisms.

Sulfur, as well as nitrogen, needs to be fixed in organic molecules with a process part of the so-called biogeochemical “sulfur cycle.” Before being incorporated into the essential organic molecules, sulfur needs to be fixed with carbon in an organic skeleton. A significant part of sulfur fixation starts in the oceans that represent a major reservoir of sulfur on Earth, with large quantities in the form of dissolved sulfate and sedimentary minerals. Inorganic sulfur, mostly SO_4_^2-^ coming from gypsum and from pyrite oxidation, is fixed by algae in the ocean upper water column to dimethylsulfoniopropionate (DMSP) [16]. DMSP produced by algae is utilized by a diverse assemblage of microbes, leading to the production of methanethiol (MeSH) and dimethylsulfide (DMS) [17, 18] (Figure 1). These compounds are highly volatile and represent a significant amount of sulfur transfer from the oceans to the atmosphere and ultimately to land. On the other hand, volcanic emissions are the main natural sources of on-land sulfur release to the atmosphere in which furthermore is oxidized via photochemical processes to various sulfur oxidation state species [19]. SO_4_^2-^, MeSH, and DMS are the most important precursors of sulfur organic compounds synthetized by plants and microorganisms [20] (Figure 1).

Cys is the precursor (thiol-reduced sulfur donor) of most organic sulfur-containing molecules in the plant metabolome. Sulfur fixation is strictly related to Cys biosynthesis in which through different enzymatic steps, oxidized sulfate, alkane sulfates, or thiosulfate are reduced to sulfide and subsequently incorporated to Cys upon Ser activation (*O*-acetylserine) [21]. Plants are also able to produce a large variety of sulfur-containing products with interesting chemical, biochemical, and pharmacological features [22–25].

**Figure 2:**
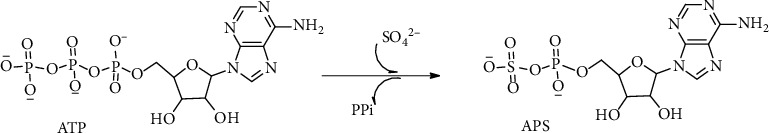
Sulfate anion incorporation in adenosine phosphosulfate (APS) from ATP.

One of the principal ways to introduce inorganic sulfur in metabolism is via SO_4_^2-^ incorporation in adenosine phosphosulfate (APS). APS is the crucial transporter of inorganic sulfur and serves as a central metabolic route for Cys biosynthesis (Figure 2) [26].

Among plant volatile organic compounds, the class of volatile sulfur compounds (VSCs) represents an important tool for plant physiology. Vegetables are one of the sources in diet for the uptake of Met and Cys, and plants possess specific metabolic pathways for the production of diverse sulfur-containing organic compounds with different important roles (defense, signaling, and communication) [27–29].

**Figure 3:**
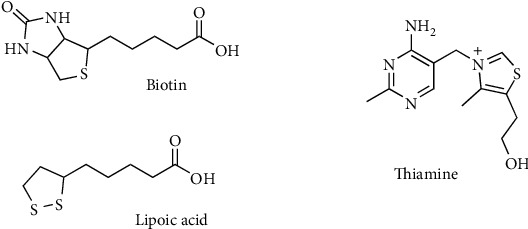
Important sulfur-containing cofactors and vitamins.

In mammals, sulfur occurs mainly in proteins as Cys and Met but also in coenzymes such as Coenzyme A (CoA), biotin, lipoic acid, and thiamine (Figure 3).

It is also common in the form of iron-sulfur clusters in metalloproteins and in bridging ligands as in cytochromes. Animals are completely dependent on preformed organic sulfur compounds to satisfy their sulfur needs [3]. Primary plant metabolism produces Met that is the essential source of sulfur for mammals and higher species such as humans. However, some higher species such as humans are able to build new sulfur-containing chemical entities starting from plants' sulfur precursors. Sulfur metabolism in humans is very complicated and plays a central role in redox biochemistry. The large number of oxidation states that this element can display makes it ideal for redox biological reaction and electron transfers (e.g., Fe-S clusters in mitochondrial proteins). Also, the biosynthesis of particular sulfur metabolites is a unique feature in some species from the animal kingdom and seems to occur via diverse biochemical pathways evolutionally far from plant sulfur metabolism [30].

The sulfur cycle, like the nitrogen cycle, is extremely important and promoted by specific prokaryotes. Most of the organic sulfur coming from this cycle is generated in the oceanic environment by microorganisms that can convert and incorporate inorganic sulfur in organic molecules necessary to satisfy the sulfur needs of all the other living beings. The metabolism of organic sulfur is at the same time a key component of the global sulfur cycle. Phototrophic and diazotrophic marine organisms such as particular marine cyanobacteria and red algae are able to use sulfur compounds as electron acceptors or donors in sulfate/sulfur reduction and oxidation [31].

## 3. Naturally Occurring Organosulfur Compounds in Terrestrial Plants and Marine Organisms

### 3.1. Natural Products from the Marine World

Marine organisms are a relatively recent successful source of novel natural bioactive compounds due also to the presence of diverse still not explored habitats and ecosystems. Several papers in the last decades reported the pharmacological activities of different compounds such as ziconotide for the management of severe chronic pain and eribulin mesylate, an antimetastatic breast cancer, both successful derivatives of compounds coming from the “marine world” [32].

#### 3.1.1. Sulfurous Amines and Amino Acids

**Figure 4:**
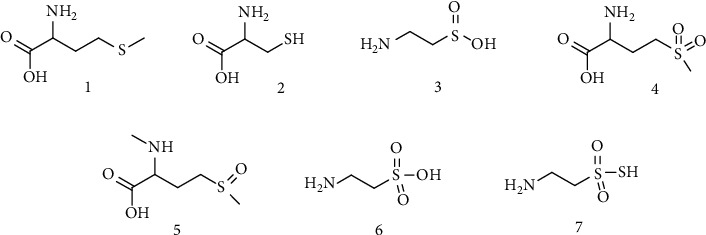
Organosulfur amines. (1) Methionine. (2) Cysteine. (3) Hypotaurine. (4) Methionine sulfone. (5) Methionine N-methyl sulfoxide. (6) Taurine. (7) Thiotaurine.

Marine microorganisms and algae are able to produce a wide range of sulfur-containing natural products, also because of the abundance of this element in the marine environment. Most of the biosynthetic routes and the biological significance of these compounds are still unknown [33]. The most ubiquitous group is that of sulfur amines and amino acids. These compounds have been found in a variety of marine organisms including algae, gorgonians, clams, and fishes, and they include Met, Cys, methionine sulfone, methionine N-methyl sulfoxide, and several aliphatic sulfonated amines (Figure 4). Studies performed on deep sea animals have detected also a high level of hypotaurine (Htau) as well as thiotaurine (Ttau) (Figure 4) [34].

These latter two sulfur amino acids seem to act as osmolytes, to balance internal osmotic pressure with that of the ocean. Ttau, in particular, seems to transport and/or to detoxify sulfide and is probably produced by the interaction of the sulfinic group of Htau with H_2_S [34–36]. The spontaneous oxidation of sulfide can produce different reactive oxygen and sulfur radical species. The presence of Ttau in these organisms is related to their need to decrease the level of sulfide; consequently, Ttau formation can be included in the mechanisms developed to counteract the presence of oxygen and sulfur radicals in the deep sea organisms. Moreover, Ttau has been proposed as a marker in animals with a sulfide-based symbiosis. This organic thiosulfate has the ability to release hydrogen sulfide (H_2_S) in a thiol-dependent reaction [37]. In particular, a thiosulfate reductase activity occurring in various cells uses electrons of thiols, such as GSH, to reduce sulfane sulfur of thiosulfonates, such as Ttau to H_2_S [38]. The H_2_S gasotransmitter is one of the most important sulfur inorganic compounds whose role is crucial in oxidative stress and inflammation processes. H_2_S has important signaling properties but also plays a crucial role in cellular redox homeostasis by modulating GSH concentration and Nrf2 factor transcription [39, 40].

#### 3.1.2. Histidine and Aromatic Derivatives

**Figure 5:**
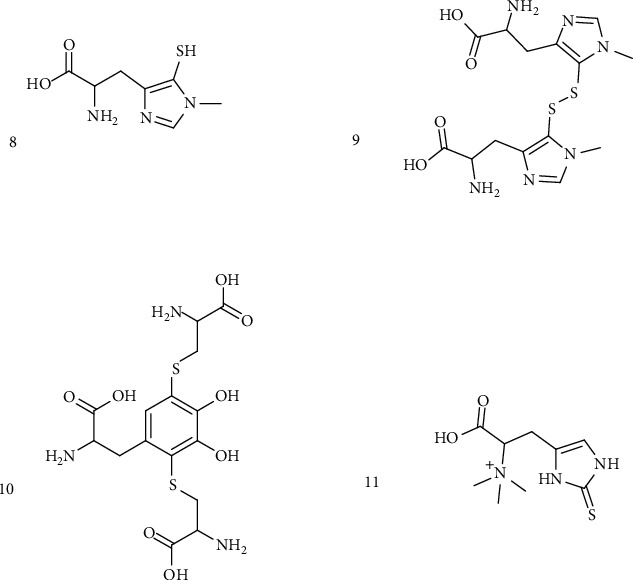
Histidine and aromatic amine organosulfur derivatives. (8) 1-Methyl-5-mercapto-L-histidine. (9) 1-Methyl-5-mercapto-L-histidine disulfide derivatives. (10) 2,5-S,S-dicysteinyldopa. (11) Ergothioneine.

Other groups of marine sulfur products are represented by the histidine derivatives and the aromatic amine derivatives. The first one includes ovothiols from echinoderm egg species such as 1-methyl-5-mercapto-L-histidine and their disulfide derivatives (Figure 5) [41], and the second includes one of the precursors of melanin produced by tyrosinase enzymes, such as 2,5-S,S-dicysteinyldopa that is part of the red-violet marine pigment, adenochrome, extracted from the branchial heart of the common octopus, *Octopus vulgaris* (Figure 5) [42]. One of the marine natural products belonging to this class of compounds that was recently found to be very promising for its biological activities is ergothioneine (Figure 5), a sulfur histidine compound derived from 2-mercapto-L-histidine by quaternization of the *α*-amino group and characterized by the presence of the sulfur thione tautomeric form [43–46]. Certain mushrooms (in the Basidiomycetes class), fungi such as *Aspergillus oryzae*, *Streptomyces* species, and cyanobacteria are the only organisms capable of producing this compound that demonstrates to possess a high pharmacological potential mainly due to its thione moiety that confers to the compound high stability and activity against ROS [47, 48].

#### 3.1.3. Indolic Compounds and Thiazolic Peptides

**Figure 6:**
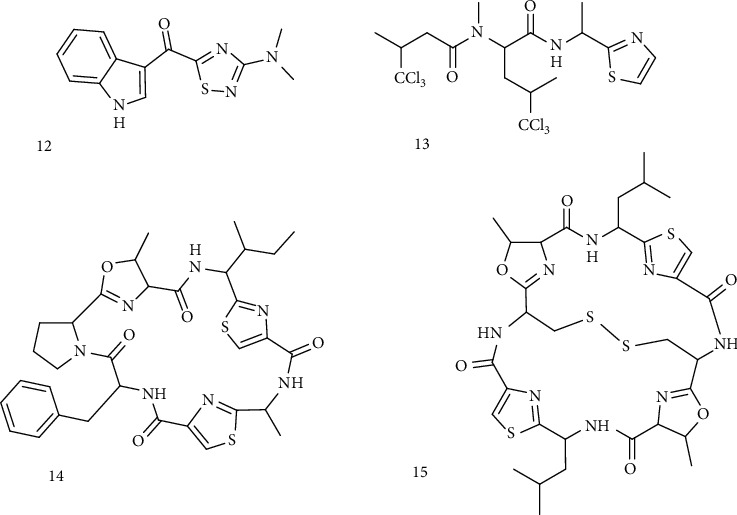
Sulfur-containing indole derivatives. (12) Dendrodoine. (13) Dysidenins. (14) Ulicyclamide. (15) Ulithiacyclamide.

Besides, indole compounds derived from tryptophan metabolism were found in marine species (Figure 6). Among this group, the occurrence of simple brominated methylthioindoles has been reported from a Taiwanese collection of the red alga *Laurencia brongniarti* (2,4,6-tribromo-3-methylthioindole and derivatives). Simple brominated methylthioindoles are encountered in the molluscan families Muricidae and Thaisidae as the precursors of the pigment Tyrian purple, used since ancient times as a valuable coloring matter. Another important indole (or guanidine) alkaloid is dendrodoine, the first identified naturally occurring 1,2,4-thiadiazole ring system extracted from a tunicate *Dendrodoa grossularia* with cytotoxic properties [49, 50]. The thiazoles and thiazolidinones group includes also dysidenin and isodysidenin pseudopeptides isolated from a collection of the marine sponge *Dysidea herbacea*. Furthermore, the tunicate *Lissoclinum patella* collected from Palau, Western Caroline Islands, gave the first two examples of thiazole-containing macrocyclic peptides, ulicyclamide and ulithiacyclamide [51].

### 3.2. Terrestrial Products

Terrestrial organisms such as plants and fungi are also able to produce an interesting pool of sulfur organic compounds.

#### 3.2.1. Alkyl and Allyl-S-Oxides

**Figure 7:**
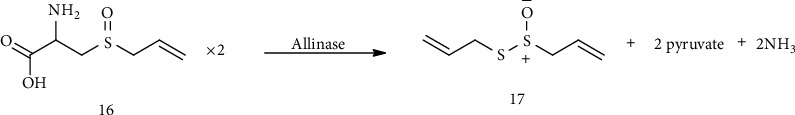
Allin (16) enzymatic conversion into allicin (17) in crushed fresh garlic.

Forms of sulfur compounds relevant to human nutrition are present in foods such as garlic, onion, and broccoli. Yet, the ingestion of these forms of sulfur compounds is very important for human health since it provides many antioxidants and immunomodulating substances that are useful to maintain an adequate physiological function of most body organs [52]. Garlic (*Allium sativum*) is the perfect example of bioactive sulfur compound (antioxidant and antibacterial molecules) intake with the diet. Allium species have a characteristic flavor that is due to the production of particular compounds when fresh garlic is crushed. When garlic is chopped, the precursors allin, isoallin, and other S-alk(en)yl-L-cysteine-S-oxides are converted via an enzymatic process mediated by allinase enzyme into the respective thiosulfinates such as allicin, isoallicin, and allysulfinates, which are also responsible for the aroma of fresh garlic and its antiseptic activity (Figure 7). Also, in onions, a series of VSCs is formed by cleavage of S-alk(en)yl cysteine sulfoxides catalyzed by allinase and lachrymatory factor synthase [53].

#### 3.2.2. Glucosinolates and Isothiocyanates

**Figure 8:**
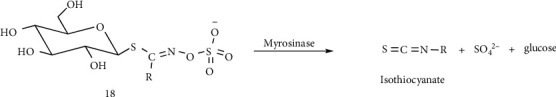
Glucosinolate (18) hydrolysis and isothiocyanate formation.

Glucosinolates are another important class of organosulfur plant secondary metabolites and are present mostly in species of the *Brassicacee* family, such as cabbage, broccoli, and horseradish, and are derived from glucose and thiohydroxamic acids starting from different amino acids [54]. Nonaromatic glucosinolates are derived mainly from Met and aliphatic amino acids, while aromatic glucosinolates, such as glucobrassicin, are derived from tryptophan as an amino acid donor. As for garlic S-alk(en)yl-L-cysteine-S-oxides, also glucosinolates represent inactive precursors that release the biologically active sulfur species when the plant material is cut, chewed, or crushed. When the enzyme myrosinase enters in contact with the glucosinolates, substrates cleave off the glucose moiety and release isothiocyanates (commonly known as mustard oil), which are responsible for pungency and defense mechanism (Figure 8) [55].

**Figure 9:**
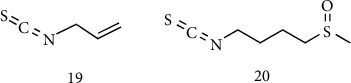
Bioactive isothiocyanates. (19) Allyl isothiocyanates. (20) Sulforaphane.

When the plant is attacked or damaged, the organism is already prepared with this two-component system (enzyme-precursor) and immediately starts the enzymatic hydrolysis of glucosinolates and the subsequent formation of the bioactive isothiocyanates [56]. Allyl isothiocyanates (Figure 9) from radish, horseradish, and wasabi are well-recognized VSCs for strong repellent activity against various arthropods, nematodes, and microorganisms and possess a good chemopreventive activity [57]. An important part of the beneficial effect of the Mediterranean diet is related to isothiocyanate intake such as sulforaphane (Figure 9). Sulforaphane, an isothiocyanate compound that occurs in high concentration as its precursor glucoraphanin, is abundant in broccoli, Brussels sprouts, and cabbages and many biological effects such as anti-inflammatory, antidiabetic, anticancer, and neuroprotective effects were recently ascribed to this compound [58].

#### 3.2.3. Polysulfides and Phytochelatins

**Figure 10:**
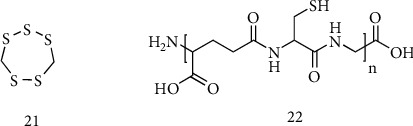
Cyclic polysulfide (21) lenthionine and (22) phytochelatins.

Other two important classes of nonaromatic sulfur derivatives are cyclic methylene-sulfur compounds (polysulfides) and phytochelatin polymers. The most famous molecule belonging to cyclic polysulfides is lenthionine. Lenthionine (Figure 10) and 1,2,4,6-tetrathiepane were earlier isolated from an extract of the edible “shiitake” mushroom (*Lentinus edodes*) and are partly responsible for its flavor. Lenthionine biosynthesis was not completely elucidated, but it seems also in this case as for garlic thiosulfinates that catalytic cleavage of C-S lyase enzyme is the crucial step for the formation of the final product [59, 60]. Phytochelatins (Figure 10) are a class of sulfur derivatives which are polymeric peptides. They are synthetized by plants, fungi, and cyanobacteria when the cellular environment is rich in metal ions. Structurally, they are polymers of GSH and their principal role is the environmental detoxification exerted by their strong activity as chelating agents [61].

## 4. Biochemical Aspects of Endogenous Sulfurous Metabolites

The metabolism of sulfur-containing amino acids consists of a variety of reactions and pathways with several intermediates and products whose biochemical significance still needs to be fully elucidated [62–66]. Crucial functions for cell survival are served by some of these metabolites. Met and Cys are the two main sulfur amino acids. They are incorporated into proteins and have important catalytic roles in the active sites of many enzymes [67, 68]. Dietary proteins normally supply Met and Cys. In addition to the intake of dietary protein, turnover of body proteins releases free Met and Cys into the body pools [69, 70].

Met serves as the source of sulfur for Cys biosynthesis in a one-way transsulfuration pathway that links metabolically Met and Cys. In mammals, Met is an essential amino acid as it cannot be synthesized in amounts sufficient to maintain the normal growth, whereas Cys is considered a semiessential amino acid because it can be produced from Met sulfur and serine via transsulfuration. Cys and Met oxidation and catabolism yield a considerable amount of energy. This was originally believed to be wasted, as the oxidation of this sulfur to sulfate (−2 → +6) was not thought to be coupled to ATP synthesis [71]. However, recent findings suggest that H_2_S derived from Cys and Met metabolism can stimulate oxidative phosphorylation via sulfide:quinone oxidoreductase (SQR) and sulfite oxidase [72–76]. Beyond this energetic potential of Cys and Met, these sulfur amino acids exert crucial functions through their well-known metabolites, such as SAM, Tau, and GSH.

### 4.1. Transmethylation/Transsulfuration Pathway

**Figure 11:**
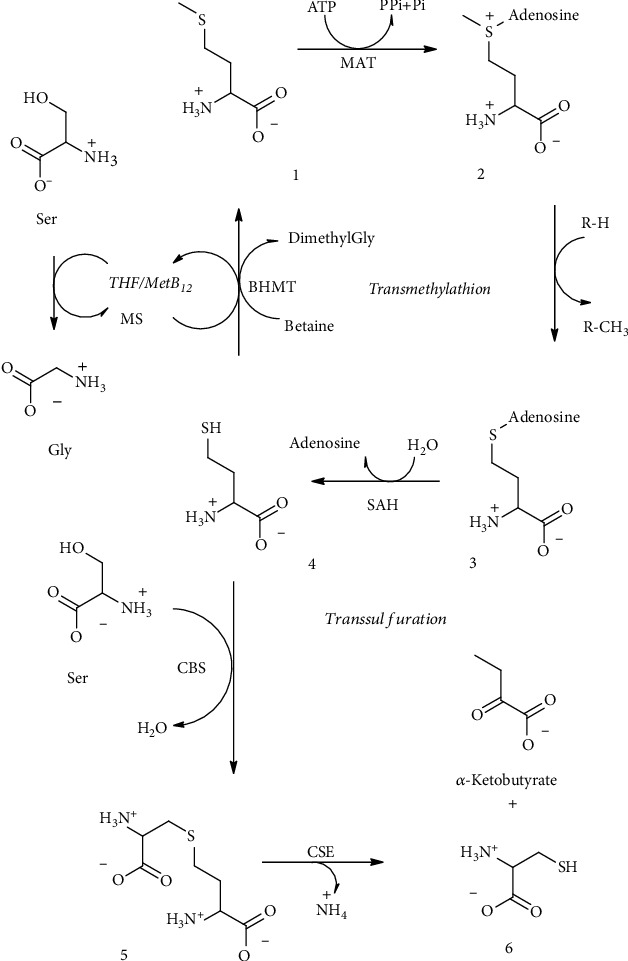
Transsulfuration/transmethylation pathway. Methionine (1), SAM (2), S-adenosylhomocysteine (3), homocysteine (4), cystathionine (5), and cysteine (6). MAT: Met adenosyltransferase; SAH: SAHCy hydrolase; MS: Met synthase; BHMT: betaine:homocysteine methyltransferase; CBS: cystathionine *β*-synthase; CSE: cystathionine *γ*-lyase.

In mammalian cells, the transmethylation/ transsulfuration pathway is central for sulfur amino acid metabolism and the regulation of redox balance. The pathway involves the transfer of sulfur from HCy to Cys via cystathionine and is the only route for biosynthesis of Cys. This pathway is intimately linked to the transmethylation pathway via HCy, which can be remethylated to generate Met or be irreversibly converted to Cys (Figure 11).

Met metabolism begins with the activation of Met to SAM by Met adenosyltransferase (MAT). The reaction requires ATP and the sequential cleavage of all its high-energy phosphate groups. SAM as a methyl group donor generates S-adenosylhomocysteine (SAHCy) by cellular methyltransferases. SAHCy is hydrolyzed to yield HCy and adenosine by SAHCy hydrolase (SAH). This sequential reaction route is present in all cell types and is referred to as the transmethylation pathway. HCy is methylated back to Met by the Met synthase (MS) and, only in the liver and the kidney of some species, by betaine:homocysteine methyltransferase (BHMT) [77]. The HCy remethylation is catalyzed by both MS and BHMT, and the combination of transmethylation and remethylation comprises the Met cycle.

The transsulfuration pathway diverts HCy from the Met cycle, converting HCy to Cys by the sequential action of two pyridoxal 5′-phosphate- (PLP-) dependent enzymes, cystathionine *β*-synthase (CBS) and cystathionine *γ*-lyase (CSE). HCy, which is derived from dietary Met, is converted to cystathionine by CBS, which is acted on by CSE to generate Cys. The transmethylation and remethylation pathway occurs in all cells, whereas the transsulfuration pathway is restricted to certain tissues. An exogenous source of Cys is required in conditions where transsulfuration reactions do not occur at a sufficient rate [78–80]. These tissues accumulate HCy (or cystathionine) which must be exported to other tissues for further metabolism/removal. The transsulfuration pathway occurs in tissues that contain both CBS and CSE [81]. CBS activity is widely present in mammalian organs including the liver, adipose tissue, kidney, and brain [82]. Conversely, it is widely assumed that a high level of CSE is present in the liver, kidney, and pancreas, whereas CSE activity is absent in the brain [83], However, recent studies have demonstrated that also CSE is both present and active in the brain [84, 85]. Both CSE and CBS occupy a central role in the cell redox regulation. It has been reported that approximately half of the intracellular GSH pool in the human liver is derived from Cys generated from HCy via the transsulfuration pathway [86]. In the mouse brain, the activity of the pathway is lower as compared to that in the liver, but the flux can be regulated by oxidative stress [84, 85]. It has been observed that CSE undergoes inactivation under oxidative stress condition in mice [65]. In this regard, an intact transsulfuration pathway plays a key role in maintaining GSH homeostasis and affords an effective neuroprotection.

### 4.2. S-Adenosylmethionine

**Figure 12:**
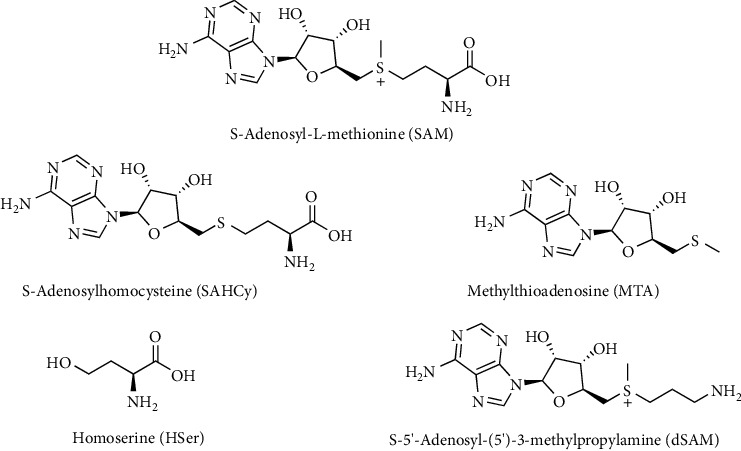
S-Adenosyl-L-methionine (SAM) degradation products.

SAM is a high-energy sulfonium compound which acts primarily as a methyl donor in reactions catalyzed by a vast array of methyltransferases. Given its high energy, the molecule is not so stable in vitro and can be degraded rapidly even at room temperature, giving rise to SAHCy, homoserine (HSer), 5′-methylthioadenosine (MTA), and S-5′-adenosyl-(5′)-3-methylpropylamine (dSAM) (Figure 12) [87–90].

These SAM-dependent methylations are essential for biosynthesis of various biomolecules including creatine, epinephrine, melatonin, carnitine, and choline. An alternative fate of SAM is decarboxylation to form dSAM, which is the donor of aminopropyl groups for synthesis of spermidine and spermine [63, 91]. As a result of polyamine synthesis, MTA forms from dSAM. Alternatively, SAM provides amino groups in biotin synthesis and 5′-deoxyadenosyl radicals and also sulfur atoms in the synthesis of biotin and lipoic acid [92–97].

SAM is also a potent allosteric regulator of the transmethylation/transsulfuration pathways. SAM promotes Met catabolism through the transsulfuration pathway and inhibits the remethylation of HCy to Met [98–101]. As a result, SAM increases the activity of CBS which is the primary enzyme in transsulfuration and contributes to the synthesis of Cys, thereby increasing the GSH level. The attenuation of oxidative stress by SAM administration has been evidenced by several studies. For example, Li et al. [102] showed that cells can be protected from oxidative stress induced by 𝛽-amyloid peptide after SAM administration; indeed, SAM actually increases endogenous antioxidant defense by restoring the normal GSH/GSSG ratio and inducing antioxidant enzyme activities.

### 4.3. Nontranssulfuration Pathway

The SAM-independent catabolic pathway of Met also occurs, involving an initial transamination reaction [62]. The transamination of Met forms *α*-keto-*γ*-methiolbutyrate, the *α*-keto acid analogue of Met, which may be further catabolized via oxidative decarboxylation to 3-methylthiopropionate, MeSH, and additional catabolites [103, 104]. This is considered a minor pathway under normal circumstances, but it becomes more significant at high Met concentrations. Because it produces powerful toxins such as MeSH, it has been considered to be responsible for Met toxicity [105]. Indeed, Met has been regarded as a toxic amino acid, whether large amounts are taken up from the diet or it accumulates from metabolic dysfunction of the transsulfuration pathway. Excessive dietary Met causes acute liver injury and erythrocyte membrane damage through mechanisms that are not still fully elucidated [104–107]. The toxic effect has been observed especially in rodent tissues where Met transamination occurs and appears to play a crucial role in Met toxicity [106]. In humans, the physiological and toxicological significance of the Met transamination pathway remains unclear [108, 109]. Interestingly, not only Met but also other thioether metabolites undergo transamination generating a class of sulfur-containing heterocyclic compounds called ketimines (described below) [110, 111].

### 4.4. Cysteine Metabolism

Cys, whether formed from Met and serine via transsulfuration or supplied preformed in the diet, serves as a precursor for synthesis of proteins and several other essential molecules. These metabolites include GSH, CoA, and Tau. These fates of Cys, except GSH, involve loss of the Cys moiety as such. Cys is a substrate for CoA synthesis in that it is used to form the cysteamine (decarboxylated Cys) moiety of the CoA molecule and, hence, contributes to the reactive sulfhydryl group. Cys is also the precursor of the gaseous signaling molecule H_2_S [112–114].

**Figure 13:**
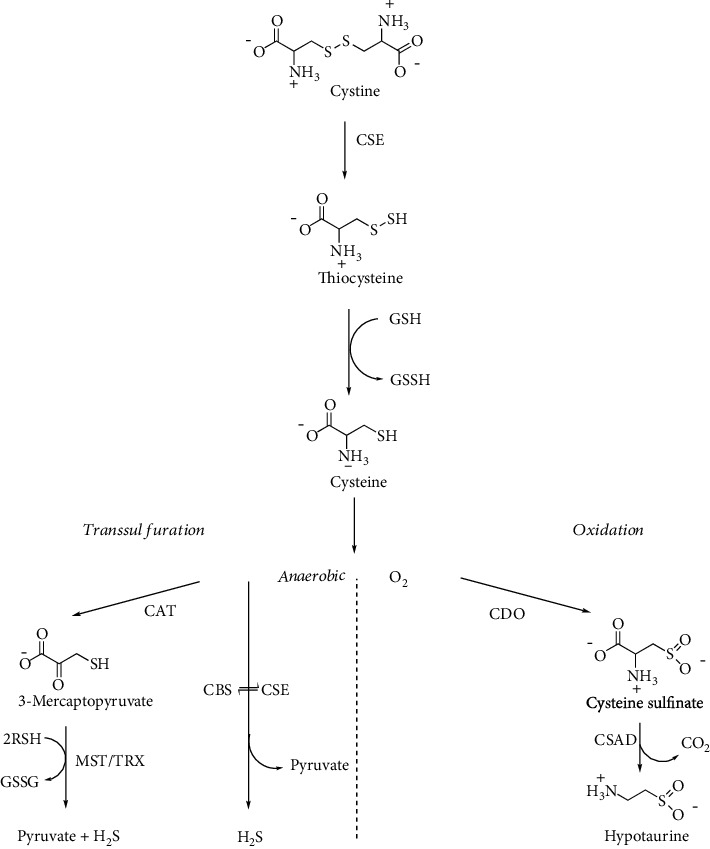
Cystine metabolism by transsulfuration enzymes. CAT: cysteine aminotransferase; CBS: cystathionine *β*-synthase; CDO: cysteine dioxygenase; CSAD: cysteine sulfinate decarboxylase; CSE: cystathionine *γ*-lyase; MST: mercaptopyruvate sulfurtransferase; TRX: thioredoxin.

Cys is metabolized via two distinct routes. The first one, called the cysteine sulfinate- (CSA-) dependent (aerobic) pathway, is a series of oxidative steps leading to Htau. The second one, a transsulfuration (anaerobic) pathway, is a source of sulfane sulfur-containing compounds as well as H_2_S [115]. The enzymes involved in the H_2_S production include pyridoxal 5′-phosphate- (PLP-) dependent CSE and CBS as well as cysteine aminotransferase (CAT) in conjunction with PLP-independent mercaptopyruvate sulfurtransferase (MST) (Figure 13) [112, 116, 117].

Cys is readily oxidized to Cys-Cys and exists in oxidized form in plasma and in the extracellular milieu, thus representing the major transport form of non-protein-bound Cys [118]. Across membranes, Cys and Cys-Cys are transported by different membrane carriers. In the CNS, glial cells mainly import Cys-Cys via the cystine-glutamate antiporter providing the major route for GSH synthesis in the brain [119]. Cys-Cys undergoes *β*-elimination reaction by transsulfuration enzymes, CBS and CSE, yielding thiocysteine, the persulfide analogue of Cys (RSSH) [120–123].

### 4.5. Glutathione

**Figure 14:**
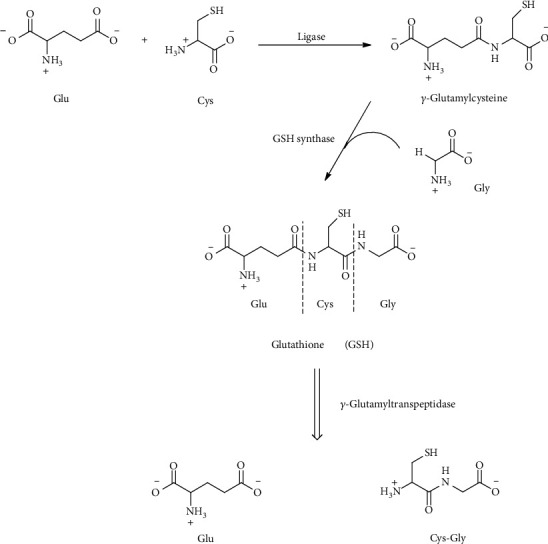
Glutathione (GSH) and its enzymatic degradation products, glutamate (Glu) and cysteinylglycine (Cys-Gly).

The GSH tripeptide is derived from Cys, glutamate, and glycine present in all mammalian cells at mM concentration level (1-10 mM) with the highest amount in the liver. GSH has an unusual *γ*-glutamyl bond linking glutamate and Cys This unconventional peptide bond through the *γ*-carboxyl group of glutamate rather than the *α*-carboxyl group confers stability to hydrolysis by cellular peptidases, requiring a specific enzyme for GSH degradation. The first step in the GSH synthesis is catalyzed by the enzyme glutamate-cysteine ligase (GCL) which forms *γ*-glutamylcysteine in an ATP-dependent reaction [124]. This conjugation reaction between glutamate and Cys is considered the rate-limiting step in GSH synthesis, whereas Cys the limiting substrate [125]. The addition of Gly to *γ*-glutamylcysteine is catalyzed by the ATP-dependent enzyme glutathione synthase (GS) which results in the formation of the mature GSH tripeptide [126, 127]. The enzyme that accounts for the hydrolysis of the *γ*-glutamyl bond is *γ*-glutamyltranspeptidase (*γ*GT), which localizes on the luminal surfaces of cells lining the glands and ducts of various organs particularly the kidney, pancreas, and liver [128] (Figure 14).

As consequence, GSH is resistant to intracellular degradation and is mainly metabolized extracellularly by cells that express *γ*GT. However, recently, cytosolic breakdown pathways for GSH have been described [129]. GSH breakdown by *γ*GT produces glutamate and cysteinylglycine which can be taken up by cells where the released amino acids are reused for the synthesis of GSH (so-called the *γ*-glutamyl cycle) [130]. In addition to the several vital functions of GSH, GSH serves as a reservoir of Cys and as a means for transporting Cys to extrahepatic tissues. An association between Cys and GSH metabolism disruption and aberrant redox homeostasis and neurodegeneration has been frequently observed [131, 132].

### 4.6. Coenzyme A, Pantetheine, and Cysteamine

**Figure 15:**
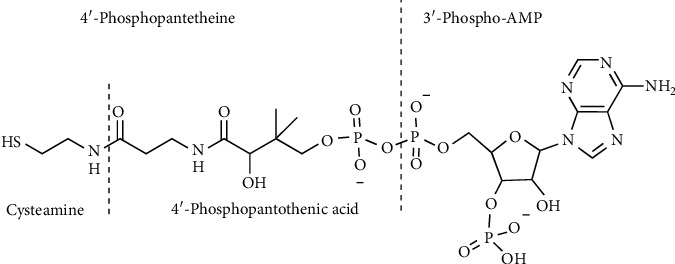
Coenzyme A. Chemical structure of CoA and its components.

**Figure 16:**
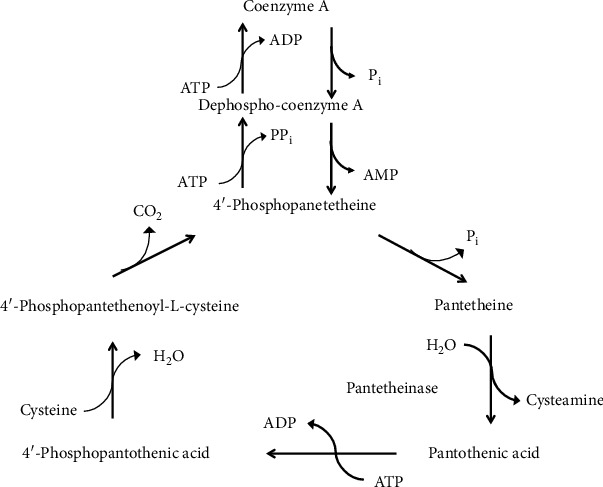
CoA biosynthesis and degradation.

CoA (Figure 15) is synthesized starting from pantothenate and cysteine in five reaction steps (Figure 16). 4′-Phosphopantetheine (cysteamine-pantothenate conjugate) is the moiety bearing the reactive thiol for the formation of the high-energy thioester bond in acetyl CoA [133].

CoA breakdown generates pantetheine which is hydrolyzed by the vanin family of pantetheinase enzymes to pantothenate and the aminothiol, cysteamine [134, 135]. Cysteamine is the decarboxylated derivative of Cys. However, in mammals, cysteamine is not formed from Cys directly by decarboxylation. Rather, it is produced mainly by the pantetheinase activity during CoA breakdown (Figure 16). An alternative route to cysteamine from lanthionine has been described, where lanthionine undergoes first decarboxylation to S-2-aminoethyl-L-cysteine (also called thialysine); subsequently, this latter compound is converted to cysteamine by a *β*-elimination reaction [136]. In humans, cysteamine undergoes different metabolic degradations such as conversion to volatile sulfur compounds, i.e., methanthiol and dimethylsulfide, which have been detected in cystinosis patients treated with this aminothiol [137, 138]. Cysteamine is highly reactive, and it readily oxidizes in solution to form the disulfide cystamine. Cysteamine readily forms mixed disulfides with susceptible Cys thiol groups in a process called cysteaminylation which is key for many reported biological activities [139]. At low concentrations, cysteamine can form a mixed disulfide with Cys, promoting Cys transport into cells. Recently, the complex role of cysteamine and cystamine as oxidative stress sensors has been illustrated by experiments using vanin 1-deficient mice [140, 141]. Interestingly, cysteamine has been used to treat cystinosis and neurodegenerative disorders [142, 143]. A recent review recapitulates the use of cysteamine as a mutation-tailored drug. This aminothiol has been proposed to repair Arg to Cys missense mutations in genetic disorders. Upon binding of cysteamine to the Cys residue by a disulfide bond, a mimic structure resembling the original arginine residue is created on the mutated protein [144].

### 4.7. Taurine

**Figure 17:**
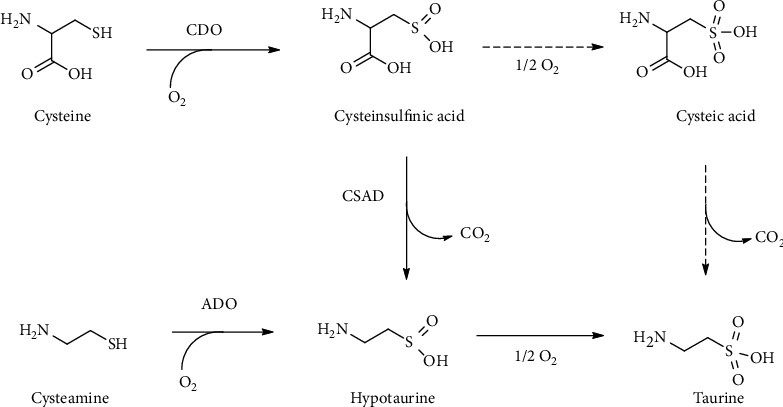
Taurine biosynthesis. Alternative routes for taurine biosynthesis from cysteine or cysteamine. ADO: cysteamine dioxygenase; CDO: cysteine dioxygenase; CSAD: cysteine sulfinate decarboxylase.

In the mammalian pathway leading from Cys to Tau, Htau is the main metabolic precursor of Tau. Htau is synthesized by the CSA-dependent (aerobic) pathway of Cys metabolism (Figure 13). The production of Htau is dependent upon the sequential action of cysteine dioxygenase (CDO) that adds molecular oxygen to the thiol group of Cys to form CSA and of cysteine sulfinate decarboxylase (CSAD) that finally generates Htau [115]. In a minor pathway, CSA can also undergo oxidation to produce cysteic acid (CA) and, through subsequent decarboxylation, forms Tau [115, 145]. Another pathway involves the production of Htau from cysteamine via the action of cysteamine dioxygenase (2-aminoethanethiol dioxygenase, ADO) (Figure 17) [146].

CDO and ADO are the only two mammalian thiol oxygenases capable of specifically oxidizing free sulfhydryl groups [147]. The activities for these two proteins were first reported in mammalian tissues almost 60 years ago [148, 149].

The importance of the cysteamine/Htau/Tau pathway has been largely regarded as minor relative to the Cys/CSA/Htau/Tau pathway. Indeed, it is the reaction catalyzed by CDO that has been implicated as the major rate-determining step in the synthesis of Htau and Tau [150]. Interestingly, the brain is capable of synthesizing Tau and yet expresses relatively little CDO [151], and it is, thus, possible that the ADO-mediated pathway is largely responsible for Htau/Tau synthesis in the brain [146].

Noteworthy, the oxidation of the sulfinic group of both Htau and CSA with production of the respective sulfonate (RSO_3_^–^), Tau and CA, is a crucial point for the generation of Tau in mammalian tissues [145, 152]. However, no specific enzymatic activity has been detected for this oxidation. Conversely, there is strong evidence that *in vivo* formation of Tau and CA is the result of sulfinate (RSO_2_^–^) interaction with a variety of biologically relevant oxidizing agents [153–156]. The relevance of both CO_3_^·–^ and ^·^NO_2_ in the oxidation of Htau has been recently evidenced by the peroxidase activity of Cu-Zn superoxide dismutase and horseradish peroxidase [157–159]. Recently, it has been shown that a wide substrate range enzyme such as flavin monooxygenase 1 (FMO1) is able to catalyze the oxidation of Htau to Tau [160].

Tau is the most abundant free amino acid in animal tissues and is present especially in excitable tissues such as the brain, retina, muscle, and heart, whereas circulating levels are, in comparison, much lower [2, 161–163]. The Tau content differs between species such that taurine levels have been reported lower in primates than in rodents. The amounts range from 2 *μ*mol/g wet weight in the human brain to 40 *μ*mol/g in the mouse heart, with even higher concentrations in the eye retina and in the developing brain of mice [164, 165]. In the muscle, retina, and neurons, the Na^+^-dependent transport through the Tau transport accumulates Tau at high levels [166]. Tau is capable of regulating osmolarity through exchange with the extracellular space without altering membrane potential. This role of Tau as a critical regulator of osmolarity is particularly important in maintaining neuronal function [167]. Both Tau and Htau exhibit neurotransmitter activity reminiscent of *γ*-aminobutyric acid (GABA) and *β*-alanine. According to this chemical structure similarity, Tau can mimic some effects provoked by GABA release [168]. In the CNS, where Tau is highly concentrated, this *β*-amino acid exhibits neuromodulator and neuroprotector activity, also preserving the homeostasis of retinal functions [169]. Tau plays a role also in bile salt formation.

Similarly, Htau is a unique amino sulfinate with a powerful antioxidant capacity [170–172]. Htau achieves a millimolar concentration in tissues and biological fluids typically subjected to high oxidative stress, such as the regenerating liver, human neutrophils, and human semen [173–175]. Noteworthy, Htau is capable of protecting SOD by the H_2_O_2_-mediated inactivation, thus reinforcing the cell defense against oxidative damage [176]. However, the one-electron oxidative reaction between Htau and various biologically relevant oxidants is accompanied by generation of reactive intermediates, such as sulfonyl radicals, which could promote oxidative chain reactions [156, 157].

### 4.8. Sulfane Sulfur: Persulfides and Thiosulfonates

Low-molecular weight RSSH such as thiocysteine and glutathione persulfides (GSSH) are present at *μ*M concentration inside the cells. Due to highly reducing and nucleophilic properties, RSSH can act as scavenging oxidants and intracellular electrophiles. RSSH are considered the major source of sulfane sulfur in biological systems. One proposed mechanism for sulfane sulfur biological effect is the modification of protein Cys residues by persulfidation [113, 177, 178]. Noteworthy, biological effects attributed to H_2_S as a signaling molecule may also be partially caused by sulfane sulfur, such as Cys-based persulfides as the actual signaling species [179–181]. H_2_S, indeed, can react with oxidized thiols, such as sulfenates (RSOH), in biological systems to give persulfides [182]. Thiocysteine can be directly generated by transsulfuration enzymes CBS and CSE from Cys-Cys, whereas GSSH is produced in the mitochondrial sulfide oxidation by SQR [40, 120, 183–185]. Recently, the *in vivo* formation of thiocysteine by the transsulfuration pathway has been questioned [186]. Sulfane sulfur species such as thiocysteine, GSSH, and protein-Cys persulfides are also generated by MST [187, 188]. Furthermore, a recent report revealed that a mitochondrial enzyme, cysteinyl-tRNA synthetase (CARS2), plays a major role in converting Cys into Cys per/polysulfide species [189].

**Figure 18:**
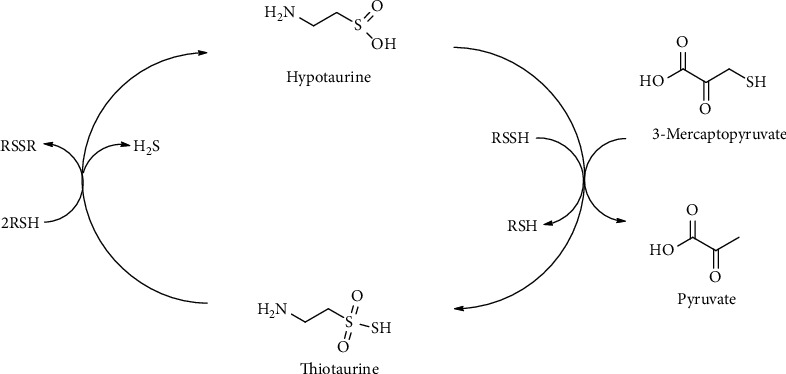
Thiotaurine transsulfuration pathway.

The sulfane sulfur of thiocysteine can be transferred by sulfurtransferases to various acceptors, including sulfite and Htau [120, 190]. The transfer of sulfur to Htau produces Ttau, a component of the Tau family characterized by the presence of a sulfane sulfur in the thiosulfonate group (−S_2_O_2_^−^). The biological occurrence of Ttau in mammals as a metabolic product of Cys-Cys *in vivo* was reported initially by Cavallini and coworkers [191] by demonstrating that rats fed with a diet supplemented in Cys-Cys excreted a newly unknown compound identified as the thiosulfonate analogue of Tau, Ttau. Furthermore, Ttau is formed by a sulfurtransferase catalyzing sulfur transfer from mercaptopyruvate to Htau [192, 193]. Structurally, Ttau carries a hypotaurine moiety and a sulfane sulfur moiety generated, respectively, by aerobic and anaerobic metabolism of cysteine. Consequently, Ttau can represent a linking intermediate of the cysteine metabolic paths (Figure 18).

Interestingly, Ttau is capable of releasing H_2_S in a thiol-dependent reaction. In particular, thiols, such as GSH, and other reducing agents reduce sulfane sulfur of thiosulfonates to H_2_S [38, 117, 193]. Accordingly, in human neutrophils, GSH acts as a catalyst in the generation of H_2_S and Htau from Ttau [194].

Overall, Ttau formed as a result of the reaction between Htau and RSSH may be converted back to H_2_S and Htau (Figure 18). It is likely that Ttau, due to its peculiar biochemical properties, takes part in the modulation and control of H_2_S signal as suggested by the effect of Ttau on human neutrophil functional responses [37, 195–197].

### 4.9. Lanthionines, Cyclic Ketimine, and Imino Acid Derivatives: Sulfur-Containing Metabolites Still in Search for a Role

**Figure 19:**
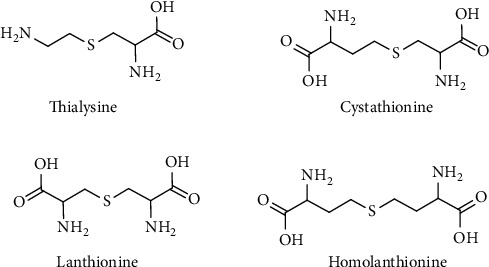
Biological thioethers belonging to the class of lanthionines

Lanthionines are a class of sulfur organic compounds that are extremely interesting but still remain a mystery. From a chemical point of view, lanthionines are thioethers derived from the condensation of aminothiols or amino acids. Cystathionine probably represents one of the most important biological thioethers for humans. This molecule is essential for the transsulfuration pathway in which HCy is converted to Cys in relation to the folate cycle and SAM-mediated methylation pathway [15]. The four most important thioethers involved in this metabolism and other mammalian metabolic disorders are lanthionine, cystathionine, S-2-aminoethyl-L-cysteine (thialysine), and homolanthionine (Figure 19).

**Figure 20:**
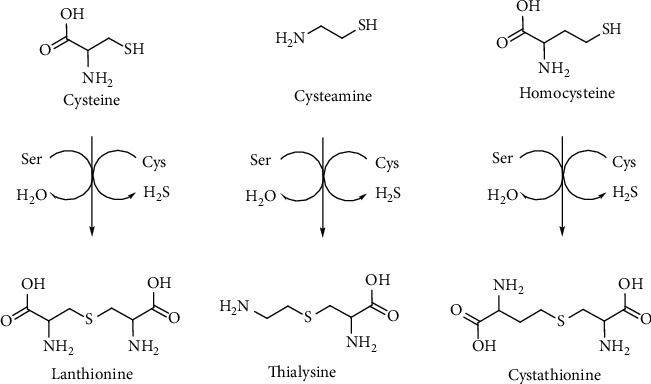
Cystathionine *β*-synthase (CBS) catalyzed synthesis of lanthionines.

The biosynthesis of these compounds in mammals is mediated by the transsulfuration enzymes, CBS and CSE, starting from Cys, cysteamine, and HCy to produce lanthionine, thialysine, and cystathionine, respectively. CBS enzyme can use serine or Cys as the substrate and releases H_2_O or H_2_S (Figure 20).

**Figure 21:**
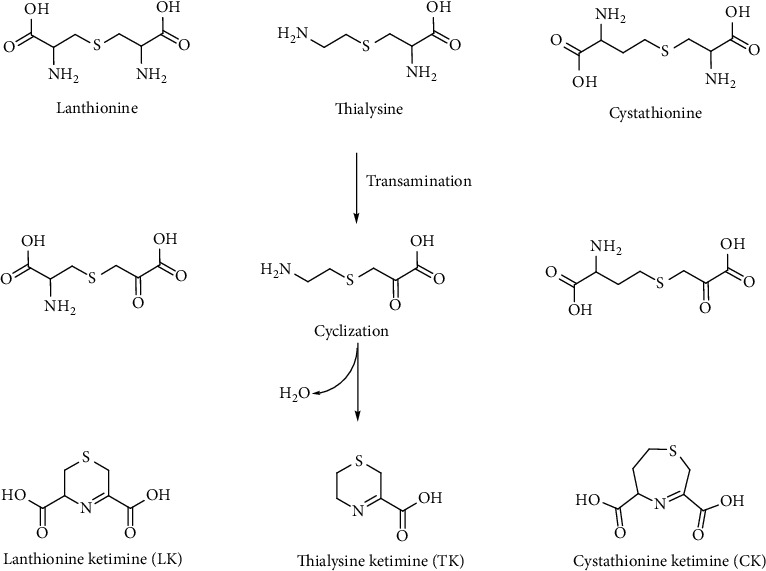
Lanthionines and related sulfur-containing cyclic ketimines generated by the brain alternative transsulfuration pathway.

The catabolism of cystathionine involves CSE releasing Cys and *α*-ketobutyric acid in a reaction that catalyzes a PLP-mediated *α*-*γ* elimination. The role of linear thioethers such as lanthionine and thialysine is still unknown from a metabolic point of view, also because they are poor substrates of CSE enzyme and then they should be addressed to different metabolic routes. Some of them such as thialysine can be used as substrates for decarboxylases giving rise to different thioether polyamines (e.g., thiacadaverine). In most of the cases, the fate and significance of lanthionine compounds is unclear and difficult to understand. In this contest, it is important to underline the fundamental contribution of professor Cavallini and coworkers in the study and discovery of some sulfur-containing cyclic ketimines as products of the linear thioethers discussed above [14]. Lanthionine, cystathionine, and thialysine can undergo an oxidative deamination to produce the respective *α*-keto acids that subsequently cyclize to give rise to their sulfur-containing cyclic ketimine, lanthionine ketimine (LK), cystathionine ketimine (CK), and thialysine ketimine (TK) [110, 198, 199] (Figure 21).

**Figure 22:**
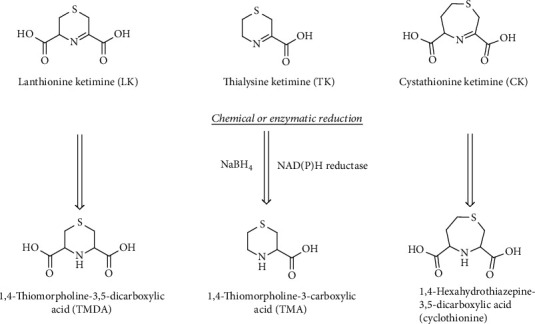
Reduced lanthionine ketimines. These compounds as their precursors were found to be produced enzymatically in specific human tissues.

LK, CK, and TK can also be obtained via a chemical reaction in aqueous solutions of Cys, HCy, and cysteamine with bromopyruvic acid [200, 201]. In the case of CK, a seven-membered asymmetric ring, the ketimine could also exist in the isomeric form with the double bond located in the longer carbon chain moiety of the ring. This isomer has been prepared by reacting *β*-chloro-L-alanine with 2-oxo-4-thiobutyrate obtained enzymatically. One common feature of this class of compounds is their reducing power. Reduced saturated cycles can be obtained by the reduction of the imine bond (C=N) with NaBH_4_ [202]. TK yields 1,4-thiomorpholine-3-carboxylic acid (TMA), LK gives 1,4-thiomorpholine-3,5-dicarboxylic acid (TMDA), and CK produces 1,4-hexahydrothiazepine-3,5-dicarboxylic acid (cyclothionine) [203]. The reduced products are much more stable than the parent ketimines and can be produced also biochemically by a NADP(H) reductase enzyme [204] (Figure 22).

All of these cyclic derivatives (LK, CK, TK, and their reduced products TMDA, cyclothionine, and TMA) were detected in human urines and mammalian brains [205–208]. LK and CK were also directly detected for the first time in the human brain in 1991 by Fontana and coworkers [209]. The very unusual and particular issue is that lanthionine differently from the other linear precursors was never found in human urines and brain tissues [208].

Lanthionines and sulfur cyclic ketimines are a very interesting class of compounds that may play an important physiological role. In degenerative processes, thiol redox biochemistry is crucial and in some neuronal disorders such as Alzheimer's disease may represent an important diagnostic marker [131]. The metabolism of organic sulfur in many organisms is still not completely understood, and there are many evidences of an emerging role of some of sulfurous ketimines in inflammation and neuronal associated disorders [15]. The brain contains a functional transsulfuration pathway able to generate H_2_S and several unusual amino acids, such as LK and other sulfur-containing cyclic ketimines and imino acids (Figure 20), whose biological role and significance are still unknown [14, 15, 210]. Among these compounds, LK demonstrated potent antioxidant, neuroprotective, and neurotrophic actions [211], properties that have made this compound a candidate for studies that focus on neurodegenerative diseases and processes including ischemia, amyotrophic lateral sclerosis, multiple sclerosis, and Alzheimer's and Batten's diseases [15, 210, 212–214].

**Figure 23:**
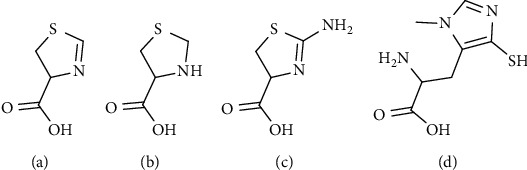
Natural heterocyclic five-membered ring compounds. (a) Thiazolin-4-carboxylic acid (derivative of formylcysteine). (b) Thiazolidine-4-carboxylic acid (thioproline). (c) 2-Amino-2-thiazoline-4-carboxylic acid (ATCA). (d) 2-Amino-3-methyl-5-sulfanylimidazol-4-yl propanoic acid (ovothiol A).

The group of natural heterocyclic sulfur compounds includes not only the six-membered ring cyclic ketimines such as CK, LK, and TK but also the class of five-membered ring heterocycles like terrestrial and marine thiazolidine and thiazoline derivatives such as thioproline, ovothiols, and thiazoline carboxylic acids (e.g., 2-amino-2-thiazoline-4-carboxylic acid (ATCA)) [41, 215–218] (Figure 23).

All of these compounds demonstrated to possess different and in some cases convergent biological activities such as oxygen and nitrogen-free radical scavenging capacity, detoxification of cyanide, and antioxidant activity [41, 217, 219–222].

### 4.10. Substrate Flexibility in the Enzymology of Sulfur-Containing Compounds

Many of the enzymes responsible of the metabolic pathways involving sulfur-containing compounds show a relaxed substrate specificity, and at the same time, many reactions involving sulfur-containing molecules are carried out by enzymes also used for different purposes.

This fact came to the attention of Cavallini and colleagues more than sixty years ago, when cystamine and lanthionamine, the R_2_S analogue of cystamine, were assayed as substrates of diamine oxidase [223, 224]. The interest of this finding was increased by the observation that the rate of the oxidation was in the range of that of traditional substrates and the product of the reaction was a cyclic cystaldimine (1,2-dehydrodithiomorpholine), which was then cleaved giving rise to a variety of products such as thiocysteamine, Htau, Ttau, and Tau.

**Figure 24:**
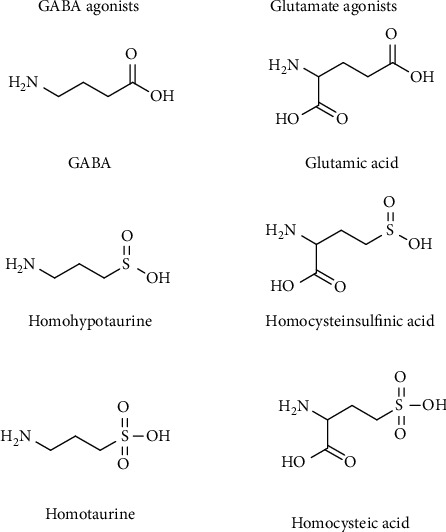
Structural relation of neuroactive sulfur amines and amino acids. These compounds can act as GABA or glutamate agonists.

CSA and CA, but also their homologues, homocysteine sulfinic acid (HCSA) and homocysteic acid (HCA), are also known to take profit from a number of enzymes used for other purposes. In this case, the sulfinic and sulfonic groups can mimic the carboxyl group of the carbon analogues aspartate and glutamate. All these sulfur compounds can substitute glutamate and aspartate in the common amino acid transamination and decarboxylation reaction. The decarboxylation reaction converts the sulfinates, CSA/HCSA, and the sulfonates, CA/HCA, in Htau/homoHtau and Tau/homoTau, respectively [225]. In addition to enzyme reactions, these metabolic derivatives interact with molecular targets such as neurotransmitter receptors, channels, or transporters. Due to structure similarity, HCSA/HCA and their decarboxylated derivatives, homoHtau/homoTau, can represent natural mimetics as neuroactive agents for glutamate and GABA, respectively (Figure 24) [226, 227].

Interestingly, the HCy derivatives can attain a major biological relevance during hyperhomocysteinemia [228]. Mild hyperhomocysteinemia is a common clinical condition associated with an increased risk for cardiovascular and neurodegenerative diseases [229, 230]. Noteworthy, in Down syndrome, or trisomy 21, the overexpression of CBS removes homocysteine from the transmethylation pathway leading to decreased plasma levels of homocysteine and a low incidence of atherosclerosis in these subjects [231, 232]. Consequently, cystathionine, cysteine, and H_2_S levels are increased, consistent with an increased CBS activity [233, 234].

In mammals, the relaxed substrate specificity makes the two transsulfuration enzymes, CBS and CSE, chiefly responsible for H_2_S biogenesis [122, 235]. These human enzymes afford H_2_S generation by a multiplicity of routes involving Cys and/or HCy as substrates. In addition to H_2_S, a variety of products is generated in these reactions, including lanthionine and homolanthionine [122]. These thioethers have been proposed as markers of H_2_S production in homocystinurias [236]. CBS is also involved in the formation of thialysine by replacing HCy with cysteamine [237]. Thialysine has been actually detected in brain tissues following gavage feeding of cysteamine in rats [238] and in urine of normal human adults, suggesting thialysine may even be a natural occurring metabolite [208].

According to this substrate flexibility in the enzymology of sulfur amino acid, a vast array of sulfur compounds occurs and can be detected in living organisms, whose biological and metabolic role is worth to be explored.

## 5. Sulfur-Containing Compounds and Redox Biochemistry

The electronic configuration of sulfur allows it to occur in numerous oxidation states, both negative and positive, ranging from -2 to +6 and possibly including fractional oxidation states [239]. Apart from the well-known ROS and RNS, the existence of reactive sulfur species (RSS) is well documented [240–242]. RSS include species such as sulfur-centered radicals (RS^·^), disulfides (RSSR), disulfide-S-oxides, and sulfenic acids (RSOH) and can easily be formed in vitro from thiols by reaction with oxidizing agents such as hydrogen peroxide, singlet oxygen, peroxynitrite, and superoxide. However, the redox potential of RSS is considerably less positive than that of ROS; their biochemical importance is not to be underevaluated. For instance, the thiyl radical is an oxidative stressor whereas the disulfide can be a mild oxidative stressor [239]. All of these RSS could in principle oxidize and subsequently inhibit redox-sensitive proteins. Furthermore, thiols could store nitric oxide via the formation of nitrosothiols, which could release nitroxyl ions [243] or nitric oxide in physiological conditions or in the presence of transition metal chelators [244–246].

The best-known RSS is thiyl radical (RS^·^), which is formed by the one-electron oxidation of Cys and is unstable in physiological conditions. If not adequately quenched by ascorbate or GSH, the thiyl radical undergoes a rather efficient intramolecular hydrogen transfer processes, and in oxidative stress conditions, the extent of irreversible protein thiyl radical-dependent protein modification increases [247]. Thiyl radical can be formed in physiological conditions via three major routes: hydrogen donation, enzymatic oxidation, and reaction with ROS. Particularly, formation of this radical has been documented for the reaction of hydrogen peroxide either with hemin or with heme proteins, such as hemoglobin [248, 249]. Many other sulfur-centered radicals could be theoretically formed, but those species are extremely unstable and can only be studied by EPR at very low temperatures; hence, their pathophysiological role, if ever, is still unclear [250]. Sulfinyl and sulfonyl radicals have also been observed as free radical metabolites of Cys oxidation, which are formed during the interaction of thiols with ROS. These species can be further oxidized to highly reactive radical species, which could lead to dimerization or oxidation [239].

Due to its high reactivity, the reduced thiol group of cysteinyl side chains in proteins plays a major role in many biological processes, and its redox state is of paramount importance in maintaining physiological functions such as catalysis, metal binding, and signal transduction. Hence, the redox regulation of the intracellular environment is a critical factor in cellular homeostasis. Particularly, the regulation of thiol redox balance is fundamental for the maintenance of many different cellular processes such as signal transduction, cell proliferation, and protein integrity and function.

The cellular thiol groups are protected by the “thiol redox buffering system,” whose key components are GSH, either reduced (GSH) or oxidized (GSSG), and the families of enzymes glutaredoxins, thioredoxins, and peroxiredoxins. GSH is the major intracellular thiol antioxidant. Apart from the antioxidant activity, GSH has also a role in the detoxification of xenobiotics and heavy metals [251]. Its concentration is in the mM range, up to 10 mM in certain cells making this compound the most concentrated antioxidant in the cells [251]. However, rather than the absolute concentration of GSH, a better index of redox state is represented by the [GSH]/[GSSG] ratio which also reflects changes in redox signaling and control of cell functions [131]. During acute oxidative stress, GSH concentration decreases and the associated increase in GSSG concentration results in an increased turnover of the GSH/GSSG cycle, but GSSG is also actively extruded from the cell; thus, the intracellular turnover of GSH is affected [251]. Under normal conditions, the ratio of GSH/GSSG is around 50 : 1 to 100 : 1, whereas in oxidative stress condition, it can drop to 10 : 1 and even to 1 : 1 [252].

Some disulfides may be strongly oxidizing and cause oxidative damage to cell components. For instance, under conditions of oxidative stress, GSSG could reach toxic concentration in the cells and oxidize proteins like metallothioneins [253]. GSSG is formed from GSH when enzymes like glutathione peroxidase (GSHPx) use GSH as a reducing species to detoxify peroxides or other ROS to prevent oxidative damage. GSH is then regenerated by the aid of NADPH in the presence of glutathione reductase (GR).

Reversible reduction of disulfide bonds can be mediated by a variety of thiol redox enzymes such as the thioredoxins (TRXs) and the glutaredoxins (GRXs). The TRX and GRX systems control cellular redox potential, keeping a reduced intracellular environment, by utilizing reducing equivalents from NADPH. These proteins are expressed in all organisms, tissues, cell types, and organelles, and some of them can shuttle between cellular compartments and the extracellular space [254].

TRXs were first identified as hydrogen donors for ribonucleotide reductase, the essential enzyme providing deoxyribonucleotides for DNA replication. The paramount importance of TRXs in the cell is witnessed by the evidence that TRX knockout is embryonically lethal [254]. There are two main forms of TRXs, TRX-1 which is present in the cytosol and TRX-2 localized in the mitochondria. TRXs are induced by oxidative stress and act as antioxidants by catalyzing the reversible reduction of disulfides utilizing both cysteinyl residues present in the active site, whose motif is Cys-Gly-Pro-Cys. Oxidized TRX is then reduced via TrxR (TRX reductase) using electrons from NADPH. As for TRXs, there are two main forms of TrxRs, one in the cytosol (TrxR-1) and one in the mitochondria (TrxR-2). Due to the easily accessible C-terminal catalytic center, TrxRs can reduce a broad range of substrates including hydrogen peroxide, selenite, lipoic acid, ascorbate, and ubiquinone, and TrxR-2 was demonstrated to act on cytochrome c [255], but the main substrates remain TRXs. In response to oxidative stress, TRXs can be secreted by the cells and exert an anti-inflammatory action by inhibiting neutrophil extravasation into the inflammatory sites, opening to the possibility of using these proteins as therapeutic tools [256, 257].

GRXs are a group of thiol redox enzymes whose active site contains the sequence motif CXXC, same as in TRXs and protein disulfide isomerases. In the GRX system, electrons flow from NADPH to GSH via GR and are then transferred to one of the three up-to-date identified GRXs. Akin TRXs, GRXs are able to reduce protein disulfides but are also able to act on mixed disulfides for which TRXs display low or no activity [258]. GRXs can act via a dithiol or monothiol mechanism, respectively, on protein disulfides or mixed disulfides, particularly on glutathionylated proteins [258]. Protein glutathionylation does not only occur in oxidative stress conditions but rather seems to be a fundamental regulatory mechanism by reversible modification of protein thiols. Hence, deglutathionylation by GRXs could represent a more general mechanism of protein activity control by GRXs than the simple regeneration of protein thiols.

## 6. Conclusions

Biomolecules consist principally of carbon, hydrogen, and the heteroatoms oxygen and nitrogen. As sulfur and oxygen belong to the same group in the periodic table, the group of chalcogens, the question that arises is as follows: “why analogue compounds with the sulfur atom replaced by oxygen do not serve the same function?”.

Sulfur actually has unique characteristics that differentiate it from oxygen, such as increased atomic size that confers to sulfur a lower electronegativity. This leads to bond formation that is less ionic and weaker than bonds between carbon and sulfur. There are also important differences in primary organosulfur metabolites with respect to oxygenated analogues, such as polarity and reactivity. In particular, thiols and thioether moieties (R_2_S) can be oxidized to sulfoxides (R_2_SO) and sulfones (R_2_SO_2_) and can form stable sulfonium cations (i.e., SAM) that allow unique carbon alkyl-transfer reactions in biology (e.g., substrates methylation). It is doubtful whether other compounds or other “-*onium*” compounds could adequately serve this role: quaternary ammonium compounds are too thermodynamically stable to effectively methylate most acceptors, and oxonium compounds are too strong alkylating agents for most of the biological environments with subsequent toxic effects.

Sulfur metabolites are utilized by all living beings and depending on the function are distributed in the different kingdoms from marine organisms to terrestrial plants and animals. Mammals, such as humans, are not able to fix inorganic sulfur in biomolecules and are completely dependent on preformed organic sulfurous compounds to satisfy their sulfur needs. However, some higher species such as humans are able to build new sulfur-containing chemical entities starting especially from plants' organosulfur precursors. Sulfur metabolism in humans is very complicated and plays a central role in redox biochemistry. In this review, we explored sulfur metabolism in relation to redox biochemistry and the large pool of naturally occurring sulfur-containing compounds in the marine and terrestrial “world.” We focused on the chemistry and the biochemistry of exogenous and endogenous sulfur metabolites underling the importance of well-known and studied molecules and also of the unknown and poorly studied sulfur natural products whose biological role is still a mystery and needs to be investigated.

It is worth investigating the role of these and other still unknown natural sulfur compounds also in view of the extremely promising beneficial activity that the molecules could exert in different pathophysiological conditions.

The biosynthesis of particular sulfur metabolites is a unique feature in some species from the animal kingdom and seems to occur via diverse biochemical pathways evolutionally far from microorganisms and plants' sulfur metabolism. It is also important to underline the somehow paradoxical situation beyond sulfur biochemistry, probably the oldest redox metabolic form of “life” on Earth and at the same time a continuously newly uncovered field with still more and more opened scientific questions.

## Acronyms

ADO: Cysteamine dioxygenase
APS: Adenosine phosphosulfate
ATCA: 2-Amino-2-thiazoline-4-carboxylic acid
BHMT: Betaine:homocysteine methyltransferase
CA: Cysteic acid
CARS2: Cysteinyl-tRNA synthetase
CAT: Cysteine aminotransferase
CBS: Cystathionine *β*-synthase
CDO: Cysteine dioxygenase
CoA: Coenzyme A
CSA: Cysteine sulfinate
CSAD: Cysteine sulfinate decarboxylase
CSE: Cystathionine *γ*-lyase
Cys: Cysteine
Cys-Cys: Cystine
DMS: Dimethylsulfide
DMSP: Dimethylsulfoniopropionate
dSAM: S-5′-Adenosyl-(5′)-3-methylpropylamine
GABA: *γ*-Aminobutyric acid
GR: Glutathione reductase
GRXs: Glutaredoxins
GSH: Glutathione
GSHPx: Glutathione peroxidase
GSSH: Glutathione persulfides
H_2_S: Hydrogen sulfide
HCA: Homocysteic acid
HCSA: Homocysteine sulfinic acid
HCy: Homocysteine
Htau: Hypotaurine
MAT: Met adenosyltransferase
MeSH: Methanethiol
Met: Methionine
MS: Met synthase
MST: Mercaptopyruvate sulfurtransferase
MTA: 5′-Methylthioadenosine
R_2_O: Ether
R_2_S: Thioether
R_2_SO: Sulfoxides
R_2_SO_2_: Sulfones
R_3_S^+^: Sulfonium ion
RDA: Recommended dietary allowance
RNS: Reactive nitrogen species
ROS: Reactive oxygen species
RS^·^: **S**ulfur-centered radicals
RSO_2_^–^: Sulfinate
RSO_3_^–^: Sulfonate
RSOH: Sulfenic acids
RSS: Reactive sulfur species
RSSR: Disulfides
−S_2_O_2_^−^: Thiosulfonate group
SAH: S-Adenosylhomocysteine hydrolase
SAHCy: S-Adenosylhomocysteine
SAM: S-Adenosylmethionine
SQR: Sulfide:quinone oxidoreductase
Tau: Taurine
TMA: 1,4-Thiomorpholine-3-carboxylic acid
TMDA: 1,4-Thiomorpholine-3,5-dicarboxylic acid
TRXs: Thioredoxins
Ttau: Thiotaurine
VSCs: Volatile sulfur compounds
*γ*GT: *γ*-Glutamyltranspeptidase.
